# Antibiotic Exposure during the Preceding Six Months Is Related to Intestinal ESBL-Producing Enterobacteriaceae Carriage in the Elderly

**DOI:** 10.3390/antibiotics11070953

**Published:** 2022-07-15

**Authors:** Man Zhang, Xiaohua Qin, Baixing Ding, Zhen Shen, Zike Sheng, Shi Wu, Yang Yang, Xiaogang Xu, Fupin Hu, Xiaoqin Wang, Yu Zhang, Minggui Wang

**Affiliations:** 1 Institute of Antibiotics, Huashan Hospital, Fudan University, and Key Laboratory of Clinical Pharmacology of Antibiotics, National Health Commission, Shanghai 200040, China; lang168968666@sohu.com (M.Z.); qxh_hs@sina.com (X.Q.); dingbaixing@126.com (B.D.); 11211300003@fudan.du.cn (Z.S.); shengzike@163.com (Z.S.); wushi68@aliyun.com (S.W.); youngyangy@163.com (Y.Y.); xuxiaogang@fudan.edu.cn (X.X.); hufupin@163.com (F.H.); 2 The National Clinical Research Center for Aging and Medicine, Huashan Hospital, Shanghai 200040, China; 3 Department of Geriatrics, Huashan Hospital, Fudan University, Shanghai 200040, China; zy_huashan@163.com; 4 Department of Laboratory Medicine, Renji Hospital, Shanghai 200127, China; 5 Department of Infectious Diseases, Ruijin Hospital, School of Medicine, Shanghai Jiaotong University, Shanghai 200025, China; 6 Clinical Epidemiology Center, Huashan Hospital, Shanghai 200040, China; wangxiaoqin@shmu.edu.cn

**Keywords:** intestinal carriage, *Enterobacteriaceae*, extended-spectrum β-lactamases (ESBLs), elderly, risk factor, infection

## Abstract

Intestinal carriage of extended-spectrum β-lactamase-producing *Enterobacteriaceae* (ESBL-PE carriage) poses a health risk to the elderly. It was aimed to study the prevalence and the risk factors of intestinal ESBL-PE carriage in the elderly. An observational study of a 921-elderly cohort was examined at health checkup for intestinal ESBL-PE carriage at a tertiary medical center in Shanghai. The prevalence and risk factors of intestinal ESBL-PE carriage, especially antimicrobial use in the preceding 9 months, were studied. The prevalence of intestinal ESBL-PE carriage was 53.3% (491/921) in community-dwelling elderly people. A total of 542 ESBL-producing isolates, including *E. coli* (*n* = 484) and *K. pneumoniae* (*n* = 58), were obtained. On genotyping, the CTX-M-9 ESBL was the most prevalent for 66.0% (358/542) of all isolates. Multivariate analysis showed that antibiotic exposure, age (61–70 years), and nursing home residence were independent risk factors of the ESBL-PE carriage. The analysis on the monthly use of antimicrobials showed that antibiotic exposure during the 6 months prior to sample collection contributed to the high prevalence of ESBL-PE carriage. A single exposure to an antimicrobial increased the risk of the carriage significantly, and the risk increased with the frequency of antimicrobial exposure (RR, 1.825 to 5.255). Prior use of second or third generation cephalosporins, fluoroquinolones, and macrolides increased the risk of the carriage. The results of this study indicate the importance of using antimicrobials judiciously in clinical settings to reduce antimicrobial resistance. Further studies with multiple center surveillance and with comparison of ESBL-PE carriage in the elderly and in the general population simultaneously are needed.

## 1. Introduction

China had a population of 138 million people aged 65 years or older, 10.1% of the total population, at the end of 2014 [1]. This proportion surpassed the World Health Organization’s definition of an aging society, i.e., people aged 60 years or older account for at least 10% of the total population. Elderly people are highly susceptible to a variety of infections, including those of the respiratory, urinary, and intestinal tract, due to multiple underlying diseases and a compromised immune system. Bacteria belonging to the family *Enterobacteriaceae* are most commonly implicated in infections of the elderly [2].

Infections caused by extended-spectrum β-lactamases (ESBLs)-producing gram negative bacilli have become more prevalent in recent years with the extensive use of third generation cephalosporins. A number of countries and regions have reported outbreaks of hospital infections caused by ESBL-producers [3]. ESBL-producers may also contribute to community-acquired infections [4,5]. They are challenging and expensive to treat. Reportedly, bloodstream infections caused by ESBL-producers are one of the most important risk factors for mortality among elderly patients [6].

Gut-colonizing *Enterobacteriaceae* constitute a reservoir of pathogens for many clinical infections, especially those related to healthcare [7]. In a recent report, for example, fecal microbiota was transplanted from a single donor to two recipients enrolled in independent clinical trials to manage recurrent or refractory *Clostridioides difficile* infection. The donor was not adequately screened for ESBL–producing *E. coli*, however, and both recipients developed bacteremia subsequent to transplantation; one recipient died. This case highlights the clinical relevance of intestinal ESBL-PE carriage and enhanced donor screening prior to fecal transplantation [8]. 

Intestinal ESBL-PE carriage in healthy elderly individuals has been little studied. Rather, most studies have focused on the prevalence of ESBL-PE carriage in nursing home residents. The reported prevalence was 3% to 11.2% in Sweden, Hungary, Belgium, and Germany [9,10], and 40.5% to 53% in Northern Ireland and Japan [10,11]. In 2014, intestinal ESBL-PE carriage was identified in 46.9% of 390 residents (≥65 years of age) in seven nursing homes in Shanghai, China [12]. It follows then that nursing home residence, prior antibiotic use, cardiovascular diseases, chronic renal insufficiency, solid tumor, Alzheimer’s disease, and repeated hospital admission significantly impact intestinal ESBL-PE carriage [13,14].

In summary, intestinal ESBL-PE carriage may seriously impair health of the elderly. The current study involved a relatively large cohort of healthy, community-dwelling elderly people living in Shanghai to study the prevalence of intestinal ESBL-PE carriage. The medical details in the preceding 9 months, especially the antimicrobial use, of the cohort were reviewed to study the risk factors of ESBL-PE carriage in the elderly.

## 2. Methods

### 2.1. Study Design and Cohort Enrollment

This study was designed as a cohort study in the elderly. The study protocol was approved by the institutional review board of Huashan Hospital. The subjects were informed of the study in detail and an oral informed consent was obtained from each participant. Elderly individuals (≥60 years of age) who came to Huashan Hospital, Fudan University for regular health checkups were sampled by anal swab (CE0123, COPAN Diagnostics Inc., Murrieta, CA, USA). Participants were assigned to the ESBL-PE carrier or noncarrier group based upon the isolation of ESBL-PE.

### 2.2. Strain Identification and Characterization

Anal swab samples were streaked onto plates composed of CHROMagar chromogenic media (CHROMagar^TM^ Microbiology Co., Ltd., Shanghai, China) that contained cefotaxime (2 μg/mL) to select for ESBL-producing isolates. The red and blue colonies on chromogenic plates were *Escherichia coli* and *Klebsiella pneumoniae*, respectively. Species were further identified using the VITEK 2 GN ID Panel and standard methods. The minimum inhibitory concentrations (MICs) of 14 antimicrobial agents for the ESBL-producing isolates were determined by agar dilution. The results of antimicrobial susceptibility testing were interpreted according to the Clinical and Laboratory Standards Institute breakpoints [15]. Intermediate (I) is a category defined by a breakpoint that includes isolates with MICs within the intermediate range that approach usually attainable blood and tissue levels and/or for which response rates may be lower than for susceptible isolates. The ESBL-producing isolates were genotyped for specific antibiotic-resistant genes (including CTX-M, SHV, and TEM) using recommended PCR primers and methodology [16]. One hundred *E. coli* isolates were selected randomly for homology analysis by multilocus sequence typing (MLST), using databases that are available at http://mlst.warwick.ac.uk/mlst/dbs/Ecoli (accessed on 25 April 2021).

### 2.3. Healthcare Details

Huashan Hospital is the only hospital dedicated to the healthcare of the study participants. All their clinic visits, in-hospital care, and regular health checkups were completed at Huashan Hospital. The medical record was reviewed to collect healthcare details for each subject in the 9 months prior to anal swab sampling. These details included age, sex, body mass index, underlying diseases, antimicrobial use in the preceding 9 months, usage of gastrointestinal modulators (gastric mucosal protective agents, probiotics, laxatives, and digestive enzymes), and prior international travel and hospital admission.

### 2.4. Statistical Analysis

SPSS software 23.0 (IBM, Armonk, NY, USA) was used for statistical analysis. Continuous variables with normal distribution were expressed as mean ± standard deviation (SD) and compared using Student’s *t*-test. A nonparametric test was used to compare the data with skewed distribution. The difference between groups was determined by the Pearson Chi-square test. Univariate logistic regression was used to analyze the risk factors for ESBL-PE carriage. The significant variables in univariate analysis (*p* ≤ 0.05) were included in multivariate logistic regression analysis to correct for confounding factors. A *p*-value less than 0.05 was considered statistically significant.

## 3. Results

### 3.1. Intestinal Carriage of ESBL-PE

ESBL-PE were carried by 491 (53.3%) of 921 community-dwelling elderly people; the remainder were noncarriers. A total of 542 ESBL-producing isolates, including *E. coli* (*n* = 484) and *K. pneumoniae* (*n* = 58), obtained from these 491 subjects were identified. Fifty-one subjects carried both *E. coli* and *K. pneumoniae*.

Of the ESBL-producing *E. coli* and *K. pneumoniae* isolates, 12.2% and 25.9%, respectively, were resistant to ceftazidime, 4.6% and 5.2%, respectively, were resistant to cefepime, and 59.5% and 55.2%, respectively, were resistant to levofloxacin; ≤1.7% of isolates were resistant to piperacillin-tazobactam or amikacin (Table 1).

On genotyping, the CTX-M-9 β-lactamase group was the most prevalent for both *E. coli* and *K. pneumoniae*, accounting for 66.0% (358/542) of all isolates. The CTX-M-9 group accounted for 66.7% (323/484) of the ESBL-producing *E. coli* isolates, followed by the CTX-M-1 group (34.9%, 169/484), the CTX-M-2 group (3.7%, 18/484), and SHV types (0.4%, 2/484). The CTX-M-9 group accounted for 60.3% (35/58) of the ESBL-producing *K. pneumoniae* isolates, followed by the CTX-M-1 (29.3%, 17/58) and CTX-M-2 (10.3%, 6/58) groups. Two isolates of ESBL-producing *E. coli* isolates but no *K. pneumoniae* isolates expressing the SHV ESBL genotype were identified, and all the isolates were detected TEM-1 positive.

MLST analysis of 100 randomly selected *E. coli* isolates identified 26 different ST types. A total of 20 isolates expressed ST131, 16 expressed ST648, 14 expressed ST1193, 7 expressed ST10, 5 expressed ST69, and 5 expressed ST48; 3 or fewer isolates expressed the other ST types.

### 3.2. Risk Factors for Intestinal ESBL-PE Carriage in the Elderly

The clinical variables of ESBL-PE carriers (*n* = 491) and noncarriers (*n* = 430) were compared (Table 2). Univariate analysis revealed that relevant to noncarriers, intestinal ESBL-PE carriage was more prevalent in the 61–70 age group than the 71–80 and ≥81 groups. In addition, antibiotic exposure, sex (female), nursing home residence, and prior international travel were risk factors for intestinal ESBL-PE carriage. Multivariate analysis showed that antibiotic exposure, age (61–70 years), and nursing home residence were independent risk factors associated with intestinal ESBL-PE carriage.

### 3.3. Effect of Antibiotic Exposure on Intestinal ESBL-PE Carriage

The monthly use of antimicrobials during the 9-month period prior to sample collection was analyzed retrospectively to explore the relationship between time of antibiotic exposure and intestinal ESBL-PE carriage. Comparing use in ESBL-PE carriers and noncarriers, the *p* values for each of the 6 months just prior to sampling were <0.001 with RR values of 2.014–3.979 (Table 3). No statistical difference between carriers and noncarriers was observed for individuals exposed to antibiotics 7- to 9-months prior to sampling. The results indicated that intestinal ESBL-PE carriage increased markedly in people exposed to antibiotics during the 6 months prior to sample collection.

A single exposure to an antimicrobial during the 9 months prior to sample collection increased the risk of intestinal ESBL-PE carriage significantly compared to no exposure (*p* < 0.001) (Table 4). Moreover, the risk of intestinal ESBL-PE carriage increased with the frequency of antimicrobial exposure (RR, 1.825 to 5.255).

Prior use of second generation cephalosporins (*p* = 0.001), third generation cephalosporins (*p* < 0.001), fluoroquinolones (*p* < 0.001), and macrolides (*p* = 0.013) increased the risk of intestinal ESBL-PE carriage (Table 5). Treatment with third generation cephalosporins had the most prominent effect (RR = 2.557).

## 4. Discussion

The present study found that intestinal ESBL-PE carriage is high (53%) in community-dwelling elderly residents. The results of this study demonstrate that antimicrobial exposure during the preceding 6 months significantly increased the risk of intestinal ESBL-PE colonization. This is a much longer period than reported in previous studies that showed antibiotic exposure only within the past 3 months or less influenced the intestinal carriage of ESBL-PE [10,11,12]. Similarly, the guidelines for hospital-acquired and ventilator-associated pneumonia states that the previous intravenous antibiotic used within the past 3 months was one of the risk factors of multi-drug resistant pathogen infections [17]. Recently, the guidance for treatment of antibiotic-resistant gram-negative bacterial infections states that clinicians should consider antibiotic exposures in the past 30 days when prescribing empiric treatment for a given patient [18]. An earlier study showed a median duration of 6.6 months (3.4–13.4-month range) for ESBL-producing *Enterobacteriaceae* carriage in inpatients [19]. Based upon the results of both the current and a few previous studies, it is recommended that infectious diseases only be treated with antimicrobials not used in the past 6 months, rather than the past 3 months.

In our previous study with a small number of participants in 2013, the intestinal ESBLs-producing *E. coli* isolates were detected in 7% (3/41) of healthy volunteers [20]. Overall prevalence of ESBLs-PE carriage in the general population in Netherland from 2013 to 2017 was 4.3% (260/5983) [21]. Therefore, it is likely the ESBL-PE carriage in the elderly is much higher than that of the general population based on this study and previous reports [10,11]. This might be attributed to more antibiotics exposure in the elderly.

The results of this study document the importance of using antimicrobials judiciously in clinical settings. Prudent antimicrobial use is the key to containing the following vicious cycle: (1) induction of multiple drug resistant (MDR) bacteria such as ESBL-PE carriage by overusing antimicrobials [10]; (2) followed by challenging, inappropriate, and long-term antimicrobial treatment of subsequent infections; and (3) emergence of more extensively drug-resistant pathogens. This study documents the influence of antimicrobial exposure on the carriage of MDR bacteria; use of antimicrobials in the prior 6 months influences MDR bacteria carriage.

There are several limitations in this study. This study was limited to a single center. The prevalence of ESBL-producing Enterobacteriaceae was only carried out in the elderly, but not in the general population simultaneously to compare the variability of ESBL-PE carriage in difference populations. Moreover, the MLST typing was performed with 100 randomly selected isolates, but not in all the 484 ESBL-producing *E. coli* isolates. It is better to identify the specific CTX-M type of all the ESBL-producing isolates rather than just the CTX-M grouping. Future studies will be undertaken to elucidate further the relevance of ESBL-PE carriage to clinical practice.

## Figures and Tables

**Table 1:** Antimicrobial susceptibility of the intestinal colonizing ESBL-producing *Escherichia coli* and *Klebsiella pneumoniae* isolates collected from elderly study participants.

Antimicrobial Agent	MIC (μg/mL)			%		
	MIC Range	MIC_50_	MIC_90_	Susceptible	Intermediate	Resistant
*E. coli* (*n* = 484)						
Ampicillin	32–>128	>128	>128	0	0	100
Cefotaxime	2–>128	32	>128	0	1.0	99.0
Ceftazidime	0.125–>128	4	32	76.0	11.8	12.2
Cefepime	0.06–>128	4	16	86.8	8.6	4.6
Cefoxitin	0.5–>128	4	16	86.6	4.4	9.0
Meropenem	0.06–0.5	0.06	0.06	100	0	0
Imipenem	0.06–0.5	0.06	0.5	100	0	0
Piperacillin-tazobactam	1–64	2	4	99.2	0.8	0
Levofloxacin	0.06–64	2	16	36.4	4.1	59.5
Amikacin	1–>128	2	4	99.2	0	0.8
*K. pneumoniae* (*n* = 58)						
Ampicillin	32–>128	>128	>128	0	0	100
Cefotaxime	0.125 –>128	32	>128	0	3.4	96.6
Ceftazidime	0.25–>128	4	32	65.5	8.6	25.9
Cefepime	0.06–>128	4	16	86.2	8.6	5.2
Cefoxitin	2–>128	4	>128	74.1	6.9	19.0
Meropenem	0.06–0.5	0.06	0.06	100	0	0
Imipenem	0.06–0.5	0.06	0.5	100	0	0
Piperacillin-tazobactam	1–64	2	4	98.3	1.7	0
Levofloxacin	0.06–64	2	16	34.5	10.3	55.2
Amikacin	1–>128	2	4	98.3	0	1.7
Fosfomycin	0.125–32	0.25	8	100	0	0

MIC = minimum inhibitory concentration.

**Table 2:** Univariate and multivariate analyses of the risk factors for intestinal carriage of ESBL-producing *Enterobacteriaceae* in the elderly.

Risk Factor	Carriers (*n* = 491) no. (%)	Noncarriers (*n* = 430) no. (%)	Univariate Analysis		Multivariate Analysis	
			*p* Value	OR (95% CI)	*p* Value	OR (95% CI)
Age, years						
61–70	100 (20.4)	41 (9.5)		1		
71–80	190 (38.7)	158 (36.7)	0.001	0.493 (0.324, 0.751)	0.001	0.419 (0.249, 0.706)
≥81	201 (40.9)	231 (53.7)	<0.001	0.357 (0.237, 0.537)	<0.001	0.312 (0.186, 0.522)
Sex (male/female)	431/60	387/43	0.001	0.427 (0.259, 0.704)	0.533	0.847 (0.503, 1.427)
Hypertension	308 (62.7)	275 (64.0)	0.701	0.949 (0.725, 1.241)		
Coronary artery disease	101 (20.6)	98 (22.8)	0.414	0.877 (0.640, 1.201)		
Respiratory tract diseases ^a^	90 (18.3)	85 (19.8)	0.579	0.911 (0.655, 1.267)		
Diabetes mellitus	136 (27.7)	95 (22.1)	0.050	1.351 (0.999, 1.827)	0.116	1.348 (0.929, 1.956)
Benign prostatic hyperplasia	55 (11.2)	48 (11.2)	0.985	1.004 (0.666, 1.514)		
Constipation	27 (5.5)	19 (4.4)	0.453	1.259 (0.690, 2.298)		
Prior surgery	95 (19.4)	81 (18.8)	0.844	1.034 (0.743, 1.437)		
Tumor	31 (6.3)	22 (5.1)	0.436	1.250 (0.712, 2.193)		
Thyroid disease	7 (1.4)	16 (3.7)	0.026	0.374 (0.152, 0.918)	0.28	0.604 (0.242, 1.506)
Prostate disease	80 (16.3)	80 (18.6)	0.356	0.852 (0.605, 1.198)		
Peptic ulcer	29 (5.9)	35 (8.1)	0.184	0.708 (0.425, 1.180)		
Liver disease	29 (5.9)	26 (6.1)	0.929	0.975 (0.565, 1.684)		
Use of gastric mucosal protective agent	104 (21.2)	85 (19.8)	0.596	1.091 (0.791, 1.504)		
Oral probiotics	62 (12.6)	60 (14.0)	0.554	0.891 (0.609, 1.305)		
Laxatives	85 (17.3)	83 (19.3)	0.435	0.875 (0.626, 1.223)		
Oral digestive enzymes	48 (9.8)	59 (13.7)	0.062	0.681 (0.454, 1.022)		
Long-term steroid use	7 (1.4)	9 (2.1)	0.439	0.677 (0.250, 1.832)		
Self-care deficit	13 (2.7)	10 (2.3)	0.755	1.142 (0.496, 2.632)		
Nursing home residence	166 (33.8)	32 (7.4)	<0.001	6.353 (4.234, 9.532)	<0.001	9.13 (5.526, 15.085)
International travel	67 (13.7)	8 (1.9)	<0.001	8.335 (3.955, 17.566)	0.088	2.112 (0.896, 4.982)
Antibiotic exposure	415 (84.5)	157 (36.5)	<0.001	9.495 (6.939, 12.992)	<0.001	11.12 (7.734, 15.989)
Hospital admission	40 (8.2)	29 (6.7)	0.420	1.226 (0.746, 2.015)		

^a^ Includes chronic bronchitis, chronic obstructive pulmonary disease, bronchiectasis, and lung cancer. OR denotes odds ratio and CI denotes confidence interval.

**Table 3:** Time of prior antimicrobial use and intestinal ESBL-PE carriage in the elderly.

Month Prior to Sampling	Antimicrobial Exposure			
	Carriers (*n* = 780) no. (%)	Noncarriers (*n* = 492) no. (%)	*p* Value	RR (95% CI)
No exposure	76 (9.7)	273 (55.5)	<0.001	0.176 (0.140, 0.221)
1st month	60 (7.7)	11 (2.2)	<0.001	3.441 (1.827, 6.479)
2nd month	108 (13.8)	28 (5.7)	<0.001	2.433 (1.631, 3.630)
3rd month	95 (12.2)	18 (3.7)	<0.001	3.329 (2.037, 5.440)
4th month	110 (14.1)	23 (4.7)	<0.001	3.017 (1.953, 4.661)
5th month	99 (12.7)	31 (6.3)	<0.001	2.014 (1.368, 2.967)
6th month	82 (10.5)	13 (2.6)	<0.001	3.979 (2.241, 7.064)
7th month	56 (7.2)	31 (6.3)	0.545	1.139 (0.746, 1.741)
8th month	45 (5.8)	28 (5.7)	0.953	1.014 (0.641, 1.603)
9th month	49 (6.3)	36 (7.3)	0.472	0.859 (0.567, 1.300)

*n*, total episodes of antimicrobial exposure during the 9 months prior to sampling; RR, relative risk; CI, confidence interval.

**Table 4:** Correlation between the frequency of intestinal ESBL-PE carriage and antimicrobial exposure during the 9 months prior to sample collection.

Frequency of Antimicrobial Exposure	ESBL-PE Carriers (*n* = 491) *n* (%)	ESBL-PE Noncarriers (*n* = 430) *n* (%)	*p* Value	RR (95% CI)
0	76 (15.5)	273 (63.5)	<0.001	0.244 (0.196, 0.303)
1	250 (50.9)	120 (27.9)	<0.001	1.825 (1.532, 2.173)
2	77 (15.7)	18 (4.2)	<0.001	3.746 (2.280, 6.155)
3	52 (10.6)	13 (3.0)	<0.001	3.503 (1.934, 6.344)
≥4	36 (7.3)	6 (1.4)	<0.001	5.255 (2.236, 12.349)

ESBL-PE = ESBL-producing *Enterobacteriaceae*; CI = confidence interval; RR = relative risk.

**Table 5:** Correlation between intestinal ESBL-PE carriage and antimicrobial exposure during the 9 months prior to sample collection.

Antimicrobials	Antimicrobials Exposure			
	ESBL-PE Carriers (*n* = 780) *n* (%)	ESBL-PE Noncarriers (*n* = 492) *n* (%)	*p* Value	RR (95%CI)
No exposure	76 (9.7)	273 (55.5)	<0.001	0.176 (0.140, 0.221)
1st generation cephalosporins	37 (4.7)	24 (4.9)	0.913	1.822 (1.269, 2.617)
2nd generation cephalosporins	104 (13.3)	36 (7.3)	0.001	2.048 (1.662, 2.525)
3rd generation cephalosporins	289 (37.1)	89 (18.1)	<0.001	2.557 (1.947, 3.359)
Fluoroquinolones	223 (28.6)	55 (11.2)	<0.001	2.334 (1.171, 4.650)
Macrolides	37 (4.7)	10 (2.0)	0.013	1.766 (0.640, 4.873)
Others	14 (1.8)	5 (1.0)	0.265	1.822 (1.269, 2.617)

ESBL-PE = ESBL-producing *Enterobacteriaceae*; *n* = sum of antimicrobial exposures during the 9 months prior to sampling; RR = relative risk.
